# Pancreatic Fistulas: Current Evidence and Strategy—A Narrative Review

**DOI:** 10.3390/jcm12155046

**Published:** 2023-07-31

**Authors:** Clara Meierhofer, Reinhold Fuegger, Matthias Biebl, Rainer Schoefl

**Affiliations:** 1 Department of Gastroenterology, Ordensklinikum Linz, 4010 Linz, Austria; 2 Department of Surgery, Ordensklinikum Linz, 4010 Linz, Austria

**Keywords:** pancreas, fistula, surgery, interventional endoscopy, drainage

## Abstract

Pancreatic fistulas are highly feared complications following surgery on or near the pancreas, abdominal trauma, or severe inflammation. These fistulas arise from leaks in the pancreatic ductal system, leading to various complications such as abscesses, delayed gastric emptying, and hemorrhage. Severe cases present with sepsis or organ failure, dramatically increasing mortality and morbidity. Risk factors include smoking, high BMI, male gender, age, and surgery-related factors like prolonged operation time and non-ligation of the main pancreatic duct. Therefore, treatment options and preventive measurements have become a hot topic in recent years. Studies have investigated the use of fibrin sealants, different closure methods, and less invasive surgical techniques. Treatment options consist of conservative measurements and the use of percutaneous drainage, prophylactic transpapillary stenting, and surgery in severe cases. As EUS has become widely available, transmural stenting started to influence the management of pancreatic fluid collections (PFCs). However, studies on its use for the management of pancreatic fistulas are lacking. Medical treatment options like somatostatin analogs and pasireotide have been investigated but yielded mixed results.

## 1. Introduction

Pancreatic fistulas belong to the most feared complications after surgery on or near the pancreas, after abdominal trauma or severe pancreatitis. The majority occur in the setting of operative interventions and are called postoperative pancreatic fistulas (POPF), with rates that vary widely from 2% to well over 20% [1].

According to the definition of the International Study Group for Pancreatic Fistula (ISGPF), they originate from a leak of the pancreatic ductal system into and around the pancreas. Furthermore, a POPF can also represent a failure of healing/sealing of a pancreato-enteric anastomosis [1]. Diagnosis is established when peritoneal drainage fluid can be measured, in which the amylase concentration exceeds the upper limit of normal serum amylase level by three times or more than 300 IU/L [1]. A variety of complications can follow, such as intra-abdominal abscess, delayed gastric emptying, and postoperative hemorrhage, as leakage of pancreatic juice can lead to vessel erosion. Systemic inflammation may result in sepsis or organ failure with an increased rate of perioperative mortality [2]. The ISGPS (updated in 2016) proposed a grading system in which POPFs are classified according to their severity. A grade A POPF has no clinical impact on the postoperative course (biochemical pancreatic leak), and no further treatment is required; however, grade B or C (clinically relevant pancreatic fistula = CR-POPF) results in a relevant change in management (intensive care unit, reoperation, and/or an extended hospital stay, etc.) with an increased risk for mortality up to 20% [2].

## 2. Risk Factors

Identifying risk factors for pancreatic fistulas has been difficult because of differences in definitions, inconsistent reporting, and low-powered studies, but they can be roughly divided into patient-related or surgery-related factors. Thus far, smoking, a high body mass index, male gender, and age have been identified to increase the risk, whereas diabetes mellitus seems to reduce it [3,4,5]. This may reflect the fact that active exocrine function is deeply involved in the development of POPF.

The surgery-related factors that have been described include prolonged operation time, excessive blood loss, the non-performance of ligation of the main pancreatic duct, transection at the tail, and additional organ resection [5]. No difference was shown after distal pancreatectomy between a soft or a hard pancreatic texture nor a higher risk for CR-POPF in patients with chronic pancreatitis [5]. When analyzing available data concerning pancreaticoduodenectomy (PD), the most reliable consensual risk factors for POPF are a small pancreatic duct (≤3 mm) and a soft pancreas tissue [4]. Distal (left-sided) pancreatectomy (DP) seems to have a higher risk for POPF as compared to pancreaticoduodenectomy since the function of the Oddi sphincter, which leads to high intrapancreatic duct pressure, is retained in the former [5].

## 3. Clinical Risk Scores

In order to identify patients at high risk for developing pancreatic fistulas (POPF), prediction models have been developed throughout the years. Among these models, the fistula risk score (FRS), alternative-FRS (a-FRS), and updated alternative-FRS (ua-FRS) have been clinically most accepted in the setting of pancreaticoduodenectomy. These models are based on intraoperative factors such as pancreatic texture and duct diameter. The original FRS included intraoperative blood loss, but its association with POPF has yielded conflicting evidence. As a result, subsequent models have excluded this variable [6,7]. However, an issue observed in the previous models is their reliance on intraoperative settings or, in some cases, postoperative factors such as histology or laboratory values as predictors. This intraoperative risk stratification approach limits the ability to engage in preoperative planning and other related considerations. Recently, several models focusing on the preoperative prediction of postoperative pancreatic fistula after pancreaticoduodenectomy have been published. Among these, only the models developed by Perri et al. and Shi et al. underwent external validation [6,8]. Perri et al. introduced a risk-tree model that relies on duct diameter and BMI, and Shi et al. presented the development and validation of a CT-based fistula risk score, which therefore requires a preoperative assessment of scans using dedicated software. Regarding AI-based prediction, calibration, and external validity were not reported in any of the available studies, resulting in limited interpretability. Prior to 2022, no prognostic models for postoperative pancreatic fistulas following distal pancreatectomy were available. Since then, De Pastena et al. created the so-called D-fistula risk score (D-FRS) [9], and Bonsdorff et al. validated the DISPAIR model [10] and introduced a predictive tool specifically tailored to this context. Both models incorporate preoperative evaluation of pancreatic characteristics derived from CT scans. In the case of the D-FRS, predictive factors include pancreatic thickness and pancreatic duct diameter measured at the neck of the pancreas. In contrast, the DISPAIR model focuses on pancreatic thickness at the transection site, the presence of transection at the neck, and whether there was preexisting diabetes. In conclusion, there appears to be a significant rise in the number of published prediction models for postoperative pancreatic fistula, but many of these models suffer from suboptimal methodology during development, lack of external validation, or unreliable performance in external settings, rendering them ineffective for practical use. Additional comparative validation is necessary.

## 4. Preemptive Strategies

Taking the morbidity of a clinically relevant fistula into account, treatment options and preventive measurements have become a hot topic in recent years.

By reviewing the literature on this topic, many studies have investigated whether fibrin sealants might influence postoperative pancreatic fistulas (POPF) in patients undergoing pancreaticoduodenectomy or if omental wrapping could reduce their incidence. Eventually, these studies failed to show a statistical difference [11,12]. Nonetheless, a recent RCT by Serradilla-Martín et al. demonstrated a significant risk reduction in clinically relevant pancreatic fistula (CR-POPF) using glycol-coated hemostatic patches, probably resuming the discussion in the future [13]. Concerning stump closure methods, there have been many publications comparing stapler versus hand-sewn closure. The multicenter randomized DISPACT trial conducted among 21 centers failed to show a superiority using a stapler closure for DP. However, a recent systematic review and meta-analysis suggested that a reinforced stapling with bioabsorbable materials in DP is safe and seems to reduce POPF grade B/C [12]. Other techniques, such as reinforcement with an absorbable fibrin sealant patch (TachoSil^®^, Takeda Austria, 4020 Linz) over the pancreatic stump or remnant pancreatic reinforcement by use of the teres ligament patch, may help to reduce POPF, but robust data are lacking. Moreover, a randomized multicenter study conducted in Austria in 2018 could not show a statistical reduction in the incidence and severity of POPF using a fibrin-coated collagen patch [11]. With respect to the advancement of minimally invasive surgical techniques, the available data concerning laparoscopic distal pancreatectomy and robot-assisted surgery indicate no significant reduction in the prevalence of postoperative pancreatic fistula (POPF). Nevertheless, these approaches show a reduced duration of exposure within the abdominal cavity and yield smaller surgical incisions, potentially contributing to enhanced perioperative results [14].

## 5. Postoperative Drains

Focusing on the routine use of intraperitoneal drain placement, they allow the evacuation of blood, pancreatic juice, bile, and lymphatic fluid. However, the time of removal, number of inserted drains, or even if an omission of drainage is possible remains unclear. There seems to be a difference between pancreaticoduodenectomy and distal pancreatectomy, considering that a POPF after pancreaticoduodenectomy can originate from underlying anastomotic dehiscence. A recently published meta-analysis concluded that no drain placement after DP was associated with a lower rate of major complications (Clavien–Dindo grade at least III), POPF, and readmissions, but limitations regarding differences in POPF definitions and or a selection bias [15] should be kept in mind. Another meta-analysis by Lyu Y. et al. showed comparable outcomes for PD and DP with or without drainage [16]. Therefore, the need for inserting a drain after pancreatic resection continues to be controversial, particularly following PD. The same holds true for the optimal timing of drain removal. When observing the existing data, the lack of an accurate definition of early drain removal seems to be predominant.

The initial randomized controlled trial comparing early drain removal (EDR) with late drain removal (LDR) was carried out by Bassi et al. [17] in patients who underwent standard pancreatic resections and were at low risk of postoperative pancreatic fistulas. Their findings indicated that a prolonged duration of drain insertion led to increased complications and longer hospital stays. Other studies, both single- and multicenter-based, by Dai et al. [18], demonstrated that EDR is safe but for selected patients. Furthermore, the American College of Surgeons’ National Surgical Quality Improvement Program (ACS-NSQIP) addressed this problem and stated that EDR following both PD and DP was associated with improved outcomes [19].

Recently, Seykora et al. concluded that early drain removal after DP is associated with a better outcome compared with late removal and no drain placement for POPF [20]. Additionally, a meta-analysis by Chen K. et al. [21] showed that EDR was associated with significantly lower incidences of grade B/C POPF and total complications for both PD and DP. Moreover, EDR was associated with a lower rate of intra-abdominal infection for PD as well as a reoperation rate and shortening of the postoperative in-hospital stay for DP. There were no significant differences in postoperative hemorrhage, biliary fistula, wound infection, intra-abdominal fluid collections, pulmonary complications, and intervention rate between EDR and LDR groups.

However, since periprocedural treatment varied between these studies, the optimal timing remains unclear even though EDR seems to be in favor. Additionally, given the risk of retrograde intra-abdominal infection that may also lead to POPF, prolonged placement of drainage should be avoided. Accordingly, percutaneous drainage further decreases the retroperitoneal pressure, which may contribute to the development of POPF because of an increasing pressure difference between the pancreatic duct and the surrounding tissue.

## 6. Medical Interventions

Concerning medical treatment options, publications have investigated the effect of somatostatin analogs as prophylactic agents to prevent POPF after pancreatic surgery. Although known for its inhibitory effects on pancreatic exocrine secretion, available meta-analyses evaluating somatostatin analogs did not demonstrate a reduction in the incidence of POPF after pancreatic surgery [22]. The recently published guidelines on the treatment of pancreatic cancer in Germany, however, propose a periprocedural somatostatin treatment in high-risk situations referring to a meta-analysis that showed a reduction in frequency and complications of POPFs [23]. Lately, pasireotide, a substance with a broader affinity to somatostatin receptor subtypes, has become more popular in the literature. Pasireotide captivates through a strong binding affinity to four out of the five somatostatin receptors, whereas octreotide specifically binds with high affinity to only two somatostatin receptors. The first RCT by Allen et al. [24] demonstrated a significant reduction in POPF after PD and DP. Nevertheless, other centers failed to prove this benefit, and a recently published meta-analysis found that prophylactic pasireotide administration after pancreatectomy was unable to decrease the incidence of POPF (clinically significant or biochemical leak) for all patients [25]. Given the high costs of these treatments, routine use in clinical practice could be difficult.

Another pharmacological approach that has emerged is the administration of perioperative hydrocortisone. Randomized clinical trials have compared hydrocortisone with placebo in high-risk patients undergoing pancreaticoduodenectomy or distal pancreatectomy, demonstrating that hydrocortisone effectively decreases major complications following pancreaticoduodenectomy and lowers the rate of postoperative pancreatic fistulas in cases of distal pancreatectomy [26]. In this setting, the anti-inflammatory effects of hydrocortisone are believed to be responsible for its mechanism in reducing complications. Nevertheless, a recently published randomized head-to-head trial failed to show a noninferiority to pasireotide in terms of reducing complications in patients undergoing partial pancreatectomy [27], and no recommendations for its administration can be made.

## 7. Stents in Pancreatic Surgery

Considering intraoperative stent placements, the available data focused on procedures where pancreaticoduodenectomies were performed, and stent placement was considered to protect the anastomosis from the corrosive effects of pancreatic juice. The stents are placed during the procedures either as internal stents or as external drainage. Studies have not shown a superiority of one method, leaving the decision up to operative habits and personal opinions. A recently published meta-analysis showed no differences in the incidence of POPF and other complications after PD but concluded that internal drainage is the optimal method to prevent POPF after PD for subgroups of patients at high risk because the surgical procedure is simple and prevents liquid loss and tube-related complications associated with external drainage [28]. Prophylactic transpapillary pancreatic stenting (PTPS) or sphincterotomy has also been proposed for the prevention of POPF, considering pancreatic duct pressure and its surrounding tissues aiming to reduce the intrapancreatic pressure, therefore, decreasing the pressure gradient along a fistula. One meta-analysis suggested that PTPS could reduce the incidence of fistulas and hospital stay after DP without increasing other complications or operative time [29]. Frozanpor et al. conducted a prospective controlled clinical trial in 2012, where the use of pancreatic duct stents before pancreatic body and tail resection did not reduce the incidence of pancreatic fistulas after pancreatic surgery. They presumed that a possible increase in the pancreatic duct pressure due to inflammation caused by the stent placement could be a factor leading to this outcome. Another clinical trial by Hackert et al. showed that the injection of botulinum toxin into the sphincter of Oddi before operation reduced the incidence of POPF after DP [30]. However, more recent data are missing, and more well-designed RCTs on this topic are needed.

## 8. Clinical Approaches

Treatment options for pancreatic fistulas should be discussed interdisciplinary and based on the updated ISGPS grading [4]. They combine conservative management strategies as already described above, endoscopic approaches, and surgery like completion of pancreatectomy as a last resort. Primary percutaneous catheter drainage is often the first intervention for severe pancreatic fistulas when no organ failure is reported. Given their minimally invasive nature, they cause less surgical trauma resulting in a fistula closure of 77.1% [31]. Nonetheless, maintaining drainage over an extended period can lead to the development of a persistent pancreatic fistula [32,33]. Patients face the potential risk of electrolyte imbalance and renal failure, which varies depending on the daily volume of fluid evacuation via the fistula. Additionally, the quality of life is negatively affected by local wound complications. Endoscopic approaches such as transpapillary interventions, e.g., stent placement across leakages or to decrease the pressure gradient at the pancreatic sphincter, are often proposed as the next step reflecting a sort of step-up approach [33,34].

The utilization of EUS-guided methods for draining peri-luminal fluid collections is not a novel concept and has now been studied for a considerable duration of time. As it has become widely available, transmural stenting started to influence the management of pancreatic fluid collections (PFCs). The role of EUS-guided PFC drainage has evolved from plastic stents to fully covered self-expandable metal stents (fcSEMS) and, recently, lumen-apposing metal stents (LAMS). However, these studies using LAMS mostly focused on pseudocysts or peri-gastric fluid collections, particularly in cases where mature wall formation led to late abscess formation or walled necrosis following acute pancreatitis. Conversely, data on its use in the management of pancreatic fistulas are still limited. In this context, they can be utilized in a manner similar to the management of pseudocysts and necrotizing pancreatitis, facilitating the drainage of fluid collections and abscesses [34,35].

Another method of using EUS-guided interventions is by performing a conversion of external drainages into internal ones via plastic stenting [32]. A recently published retrospective data analysis by Felsenstein et al. [36] demonstrated the practicality, effectiveness, and safe implementation of transgastric perianastomotic plastic stenting for internal drainage of fluid collections following pancreatoduodenectomy. They observed an earlier resolution and reduced hospitalization for patients with anastomotic leakages, aligning well with modern enhanced recovery after surgery (ERAS) concepts that facilitate prompt access to adjuvant treatments for oncology patients. In this cohort, EUS-guided drainage was used in almost one-third of the documented CR-POPF as early postoperative treatment, even in the absence of abscesses.

A large multicenter retrospective case series by Jürgensen et al. [32] showed that as far as pancreatic fistulas are concerned, there is a significant difference in terms of the presence or absence of a pancreatic fluid collection (PFC). They stated that patients who have an abdominal fluid collection are at increased risk of infection, sepsis, vascular erosion, and progressive necrosis. Those without fluid collections are comparatively more stable, presenting with lower systemic infection indicators and allowing for a more cautious and deliberate approach. For patients with pancreatic fluid collections, they noticed a considerably faster clinical improvement with a high technical and clinical success rate for EUS-guided drainage in comparison to a percutaneous approach. This endoscopic-based method also demonstrated a better tendency, although not statistically significant, in primary resolution and a favorable mortality rate. Nevertheless, the EUS-guided approach was only used in 29% of the treated individuals, and in the majority of patients, a plastic pigtail stenting was used. It is worth noting that in refractory cases, EUS demonstrated a high efficacy of 96% as a rescue approach.

Despite the inclusion of several tertiary referral centers in this data analysis, the internalization of a persisting fistula as a treatment modality was only performed in 10 patients without fluid collections. In this particular context, the primary success rate between EUS-guided internalization and remaining surgical drainage was not significantly different; only the time until fistula resolution showed a superiority towards internalization. Surgical interventions such as reoperation or potentially total pancreatectomy should be reserved for patients who are not candidates for a minimally invasive intervention or whose condition is progressively worsening with catheter drainage. There is no agreement on how to use pharmacological treatments, such as somatostatin analogs, when fistulas are already present but enteral nutrition, as well as anti-infection treatments if any signs of bacterial inflammation are evident, should be encouraged [37]. The choice of the treatment approach is currently not standardized and relies on the specific expertise and practices of each center as well as patient-related factors, including age, comorbidity, the presence of sepsis, and postoperative situs.

In clinical practice, endoscopic ultrasound (EUS) is mostly used as a secondary intervention or in refractory cases. However, recent findings suggest that EUS could be a viable alternative for the primary treatment of postoperative pancreatic fistulas, potentially resulting in a quicker resolution.

## 9. Conclusions

By observing all these different aspects of pancreatic fistulas, it appears that an adequate pre- and intraoperative risk evaluation should be implemented, and treatment options should favor a minimally invasive approach when clinically relevant fistulas are present. Nevertheless, this “hot” topic will continue to be a subject of scientific discourse.

## Data Availability

Data were obtained using the references.

## Abbreviations

ISGPF: Study Group for Pancreatic Fistula, BMI: body Mass Index, POPF: postoperative pancreatic fistulas, CR-POPF: clinically relevant pancreatic fistula, DP: distal (left-sided) pancreatectomy, PD: pancreaticoduodenectomy, FRS: fistula risk score, EDR: early drain removal, LDR: late drain removal, PTPS: prophylactic transpapillary pancreatic stenting, EUS: endoscopic ultrasound, PFC: pancreatic fluid collections, fcSEMS: fully covered self-expandable metal stents, LAMS: lumen apposing metal stents.
